# Generation of 2′,3′-Cyclic Phosphate-Containing RNAs as a Hidden Layer of the Transcriptome

**DOI:** 10.3389/fgene.2018.00562

**Published:** 2018-11-27

**Authors:** Megumi Shigematsu, Takuya Kawamura, Yohei Kirino

**Affiliations:** Computational Medicine Center, Sidney Kimmel Medical College, Thomas Jefferson University, Philadelphia, PA, United States

**Keywords:** 2′, 3′-cyclic phosphate (cP), cP-containing RNA (cP-RNA), cP-RNA-seq, ribonuclease, tRNA half, non-coding RNA (ncRNA), angiogenin (ANG)

## Abstract

Cellular RNA molecules contain phosphate or hydroxyl ends. A 2′,3′-cyclic phosphate (cP) is one of the 3′-terminal forms of RNAs mainly generated from RNA cleavage by ribonucleases. Although transcriptome profiling using RNA-seq has become a ubiquitous tool in biological and medical research, cP-containing RNAs (cP-RNAs) form a hidden transcriptome layer, which is infrequently recognized and characterized, because standard RNA-seq is unable to capture them. Despite cP-RNAs’ invisibility in RNA-seq data, increasing evidence indicates that they are not accumulated simply as non-functional degradation products; rather, they have physiological roles in various biological processes, designating them as noteworthy functional molecules. This review summarizes our current knowledge of cP-RNA biogenesis pathways and their catalytic enzymatic activities, discusses how the cP-RNA generation affects biological processes, and explores future directions to further investigate cP-RNA biology.

## Introduction

**FIGURE 1:**
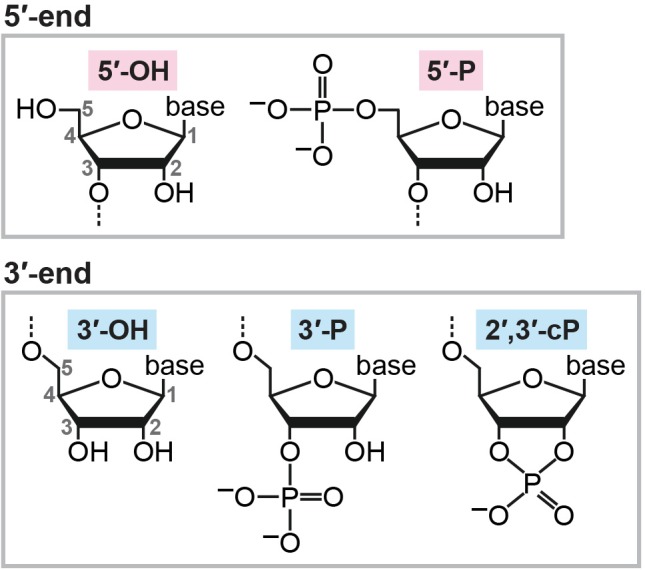
Chemical structures of RNA termini.

After transcription, newly synthesized RNA molecules must undergo maturation steps to become functional molecules, and unnecessary RNAs are subjected to turnover. In both the RNA maturation and turnover mechanisms, enzymatic cleavage of RNA molecules plays a crucial role. When cleaved, RNAs can generally possess a hydroxyl group (OH), a phosphate (P), or a 2′,3′-cyclic phosphate (cP) at their termini. While OH and P can be found at both the 5′- and 3′-ends of RNAs, a cP presents only at the 3′-end of RNAs in which the 2′- and 3′-positions of ribose is bridged by the phosphate (Figure 1). Catalytic machineries of RNA cleavage determine the terminal phosphate states of the generated RNA molecules, which is not just a consequence of the cleavage, but, in many cases, is critical for further RNA maturations and functions. The current, standard RNA-sequencing (RNA-seq) methods rely on 5′-P/3′-OH ends of RNAs, and thus, RNAs with a cP (cP-containing RNAs: cP-RNAs) cannot be captured because cP end cannot be ligated to the 3′-adapter by ATP-dependent ligase. Consequently, cP-RNAs are “invisible” in RNA-seq data and therefore form a hidden component of transcriptome. However, accumulating evidence indicates that the cP-RNA generation is significant in various biological processes. Here, we summarize our knowledge of cP-RNAs’ biogenesis mechanisms, expression, and molecular functions and discuss how to further interrogate cP-RNA biology.

## Possible Catalytic Mechanisms of cP Formation

**FIGURE 2:**
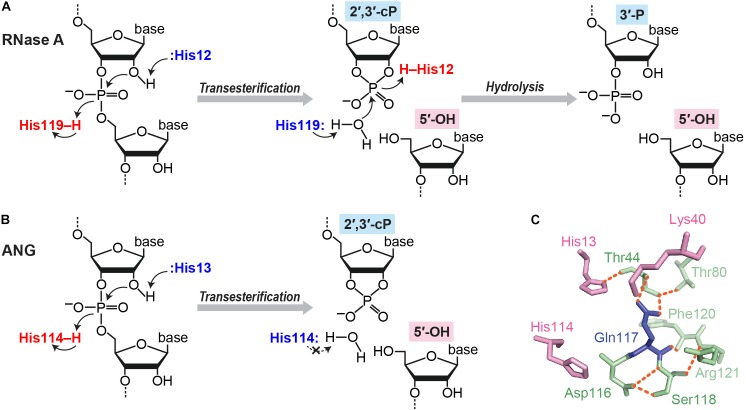
cP formation via RNA cleavage by ribonucleases. **(A,B)** Cleavage reactions catalyzed by RNase A **(A)** and ANG **(B)**. **(C)** Structure of ANG catalytic site (human wild type, PDB ID: 1B1I). Three catalytic residues are shown in pink, and the residues forming a substrate binding pocket and/or associating with Gln117 are shown in green. Orange dotted lines indicate the hydrogen bonds that particularly support Gln117’s obstructive position (Leonidas et al., 1999, 2002; Holloway et al., 2004).

There are multiple situations in which a cP is formed at the 3′-end of RNA molecules. cP frequently appears as an intermediate form during RNA cleavage by many endoribonucleases [e.g., pancreatic ribonuclease (RNase A), RNase T_1_, and RNase T_2_], which eventually generate RNAs with 3′-P/5′-OH ends (Cuchillo et al., 1997; Irie, 1997; Nichols and Yue, 2008). RNA cleavage by these enzymes is composed of two steps: (i) transesterification (transphosphorylation), forming an intermediate cP, and (ii) cP hydrolysis to generate a 3′-P (Fabian and Mantsch, 1995; Lilley, 2011) (Figure 2A). RNase A, the best studied enzyme among such endoribonucleases, contains a catalytic triad, His12, Lys41, and His119, which, especially the two histidines, serve as general acid-base catalysts during both steps (Roberts et al., 1969; Thompson and Raines, 1994; Cuchillo et al., 2011). Step (i) is initiated with 2′-OH deprotonation by a base catalyst, His12, followed by nucleophilic attack of the phosphorus by the generated 2′-oxygen (O), which causes transesterification to form a 2′,3′-cP. His119 assists the reaction as an acid catalyst by donating a proton to the leaving group, forming a 5′-OH end. Lys41 forms a hydrogen bond with 2′-O to transiently stabilize the cP. In step (ii), to hydrolyze the cP, His119 serves as a base catalyst to remove a proton from the vicinal water molecule, while His12 serves as an acid catalyst by donating a proton to form 2′-OH, generating a 3′-P as a final form.

Although the above case produces a cP just as an intermediate form, many ribonucleases, as summarized in Table 1, generate a cP as a final form via their RNA cleavage that only conducts step (i) without proceeding to step (ii) (Figure 2B). As a well-studied example, RNA cleavage by angiogenin (ANG), an endoribonuclease belonging to the RNase A superfamily (Dyer and Rosenberg, 2006; Sheng and Xu, 2016), yields a cP end (Shapiro et al., 1986). ANG contains the catalytic triad, His13, Lys40, and His114, which are well-conserved among RNase A superfamily members, but shows 10^5^–10^6^-fold lower ribonucleolytic activity compared to RNase A (Shapiro et al., 1986; Harper and Vallee, 1989). Certain unique structural features of ANG can explain this low catalytic activity. ANG’s substrate binding pocket is obstructed by Gln117 (Acharya et al., 1994; Russo et al., 1994) (Figure 2C), which is stabilized by a hydrogen bond with Thr44 (Leonidas et al., 1999, 2002; Holloway et al., 2004). Two hydrogen bonds from Asp116 and Ser118 further stabilize Gln117’s obstructive position (Russo et al., 1994). These steric hindrances would cause decreased substrate accessibility, possibly leading to low cleavage and cP hydrolysis activities. Indeed, a single mutation of Gln117 to Gly showed at least a ∼20-fold increase in ribonuclease activity, as well as a ∼28-fold increase in cP hydrolysis activity (Russo et al., 1994; Leonidas et al., 2002). In addition, Asp116 could contribute to low cP hydrolysis activity as well as low cleavage activity. While the corresponding Asp121 of RNase A forms a hydrogen bond with catalytic His119, presumably to support its imidazole ring orientation, Asp116 of ANG does not support catalytic His114 but forms two hydrogen bonds with Ser118 (Leonidas et al., 2002). The lack of support for His114 should have an adverse effect on the cP hydrolysis reaction because His114 would have initiated the reaction as a base catalyst, possibly leaving a cP as a final form.

**Table 1:** Ribonucleases reported to generate a cP as a final form.

Ribonuclease	Type	cP-examined organism	Target RNA	Physiological role	Reference validating the cP formation
ANG	Endo	Human	Mature tRNAs	Production of tRNA halves (tiRNAs, SHOT-RNAs)	Shapiro et al., 1986; Honda et al., 2015
IreI	Endo	Human, *S. cerevisiae*	*XBP1 (HAC1)* mRNA	Xbp1 mRNA splicing in the UPR pathway	Gonzalez et al., 1999; Shinya et al., 2011
PP11	Endo	Human	(not identified)		Laneve et al., 2008
Sen2	Endo	*S. cerevisiae*	Precursor tRNAs containing an intron	tRNA splicing	Peebles et al., 1983; Trotta et al., 1997
Las1	Endo	*S. cerevisiae*	37S precursor rRNA	rRNA maturation	Gasse et al., 2015
GCN4	Endo	*S. cerevisiae*	(not identified)		Nikolaev et al., 2010
zymocin (γ-subunit)	Endo	*K. lactis*	tRNAs-GluUUC, LysUUU, GlnUUG	Toxin to inhibit other yeasts’ growth	Lu et al., 2005
PaT (PaOrf2)	Endo	*P. acaciae*	tRNA-GlnUUG, GlnCUG	Toxin to inhibit other yeasts’ growth	Klassen et al., 2008
MazF	Endo	*E. coli*	mRNAs, tRNAs, rRNAs	TA system	Zhang et al., 2005a
ChpBK	Endo	*E. coli*	mRNA	TA system	Zhang et al., 2005b
Colicin E5	Endo	*E. coli*	tRNAs-TyrGUA, HisGUG, AsnGUU, AspGUC	Toxin to kill other bacteria	Ogawa et al., 1999, 2006
Colicin D	Endo	*E. coli*	tRNAs-ArgACG,ArgCCG, ArgUCU, ArgCCU	Toxin to kill other bacteria	Tomita et al., 2000
prrC	Endo	*E. coli*	tRNA-LysUUU	Host defense upon phase infection	Amitsur et al., 1987
NendoU	Endo	Nidovirus	(not identified)		Ivanov et al., 2004
Type IB topoisomerase	Endo	Vaccinia virus	(not identified)		Sekiguchi and Shuman, 1997; Shuman, 1998
USB1 (MPN1)	Exo	Human	U6 snRNA	U6 snRNA maturation	Mroczek et al., 2012

*Nomenclatures of ribonucleases are according to the organisms examined and reported. PP11, placental Protein 11; GCN4, general control protein 4; Endo, endoribonuclease; and Exo, exoribonuclease*.

RNA cleavage by colicin E5, a cytotoxic endoribonuclease found in *Escherichia coli*, also yields a cP as a final form (Ogawa et al., 1999; Ogawa et al., 2006), presumably due to cP structure stabilization. The ribonuclease domain of colicin E5 does not contain histidines, the most frequently utilized catalytic residues (Bartlett et al., 2002), but possesses Arg33 and Lys25 (numbering from C-terminal domain) as catalytic residues (Yajima et al., 2006; Inoue-Ito et al., 2012). Although the catalytic mechanism remains to be further examined, these residues, along with Ile94 that supports the orientation of Arg33, might stabilize a cP structure (Inoue-Ito et al., 2012), which would contribute to generating a cP as a final form. A cP structure may also be stabilized through interaction with a protein during RNA cleavage of a eukaryotic cP-forming exoribonuclease, U six biogenesis protein 1 (USB1), also known as mutated in poikiloderma with neutropenia protein 1 (MPN1). USB1 contains two well-conserved His-x-Ser (HxS) catalytic motifs in the active site cleft (Mroczek et al., 2012; Hilcenko et al., 2013). It is speculated that, while His120 and His208 in these motifs serve as general acid-base catalysts, Ser122 and Ser210 in these motifs coordinate the oxygens in a cP after transesterification, potentially stabilizing a cP structure as a final form by preventing further hydrolysis.

While cP end is predominantly formed by ribonuclease-catalyzed transesterification, RNA 3′-terminal phosphate cyclase (RtcA) can catalyze *de novo* cP formation by a distinct molecular mechanism involving the following three steps (Genschik et al., 1997, 1998; Billy et al., 2000; Filipowicz, 2016). First, RtcA is autoadenylylated with ATP to form a covalent RtcA-AMP intermediate. The autoadenylylation is initiated by a His309 (in *E. coli* RtcA; His320 in human RtcA)-mediated nucleophilic attack of ATP α-phosphorus, followed by covalent bond formation and pyrophosphate (PPi) release. Second, the holoenzyme then transfers the AMP to 3′-P of the substrate RNA to form an RNA with 3′-PP-5′A. Third, the energetically unstable phosphoanhydride bond between the two phosphates is cleaved by 2′-OH-mediated attack, resulting in cP formation and releasing AMP.

## cP-Forming Enzymes

### Ribonucleases

**FIGURE 3:**
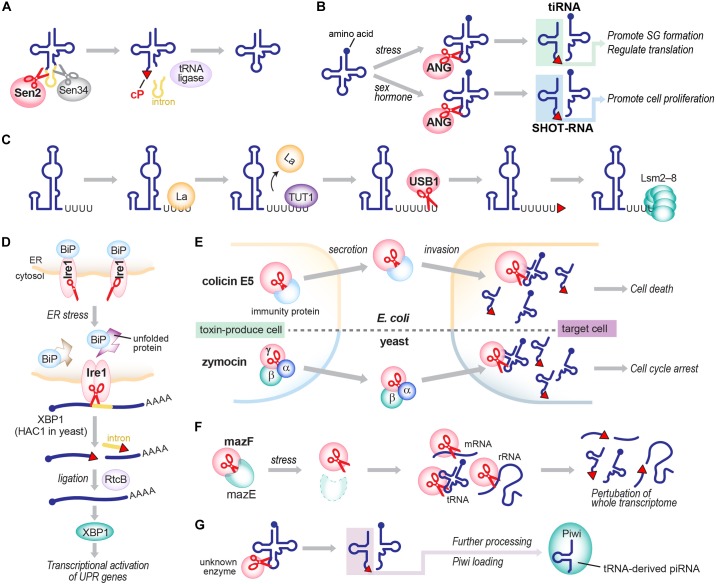
Generation of cP-RNAs in tRNA splicing **(A)**, tiRNA/SHOT-RNA production **(B)**, U6 snRNA maturation **(C)**, the UPR pathway **(D)**, toxin invasions **(E)**, the TA system **(F)**, and the biogenesis of tRNA-derived piRNAs **(G)**.

Although the detailed molecular basis of cP formation remains to be determined, RNA cleavage by many ribonucleases produces a cP as a final, predominant form, generating cP-RNAs (Table 1). A tRNA splicing endonuclease is one of the oldest ribonucleases known to generate cP-RNAs (Abelson et al., 1998; Hopper and Phizicky, 2003; Yoshihisa, 2014). In eukaryotes, precursors of some tRNAs, such as tRNA^LeuCAA^, tRNA^IleUAU^, and tRNA^TyrGUA^, contain an intronic region within their anticodon-loop (Chan and Lowe, 2016). Although the splicing activity to remove tRNA introns and cP formations during the splicing were discovered in the early 1980s (Peebles et al., 1983), many years and much effort were required to identify tRNA-splicing endonuclease subunit 2 (Sen2) as the endoribonuclease directly responsible for tRNA splicing (Trotta et al., 1997; Paushkin et al., 2004; Phizicky and Hopper, 2010), partly due to its membrane association property and low cellular expression level. Sen2 is a subunit of the heterotetrameric SEN complex and cleaves the 5′-splice site of tRNAs to leave a cP at the 3′-end of 5′-exons (Figure 3A), whereas the 3′-splice site is cleaved by Sen34. As expected from its crucial role in tRNA splicing, *SEN2* is an essential gene in yeast (Trotta et al., 1997). In humans, *SEN2* gene mutations are associated with pontocerebellar hypoplasia (Budde et al., 2008; Namavar et al., 2011; Bierhals et al., 2013).

Angiogenin, originally identified as a protein factor promoting angiogenesis (Fett et al., 1985; Kurachi et al., 1985; Strydom et al., 1985), is another ancient enzyme that produces cP-RNAs (Shapiro et al., 1986). ANG has diverse physiological roles and is associated with various pathological conditions such as cancers and neurodegenerative diseases (Tello-Montoliu et al., 2006; Gao and Xu, 2008; Sheng and Xu, 2016). tRNAs were identified as major endogenous RNA targets of ANG in *Xenopus* oocytes (Saxena et al., 1992), and, subsequently, ANG-mediated cleavages of tRNA anticodon-loops were reported to generate functional tRNA half molecules in human cell lines (Fu et al., 2009; Yamasaki et al., 2009; Honda et al., 2015) (Figure 3B). In hormone-dependent cancer cells, ANG cleavage has been shown to occur for mature aminoacylated tRNAs, generating 5′-tRNA halves with a 5′-P and a 3′-terminal cP, and 3′-tRNA halves with a 5′-OH and a 3′-terminal amino acid (Honda et al., 2015). Although *ANG* homologs are only found in vertebrates (Cho and Zhang, 2006; Sheng and Xu, 2016), 5′-tRNA halves expressed in *Bombyx mori* cells still contain a cP (Honda et al., 2017), suggesting that, even in the absence of *ANG* homologs, those organisms express an unidentified cP-forming endoribonuclease to cleave tRNAs and generate tRNA halves as cP-RNAs.

In vertebrates, U6 snRNA mostly contains a cP (Lund and Dahlberg, 1992), whereas all other snRNAs do not (Maraia and Intine, 2002). During maturation of U6 snRNA, several uridines are added to the 3′-end of a precursor RNA by terminal uridylyl transferase 1 (TUT1). Subsequently, USB1 (also known as MPN1), a cP-forming 3′ to 5′ exoribonuclease, excises a 3′-terminal uridine stretch to generate a mature 3′-end with four or five uridines containing a cP (Shchepachev et al., 2012; Mroczek and Dziembowski, 2013; Didychuk et al., 2018) (Figure 3C). Although USB1 belongs to the 2H phosphoesterase superfamily and contains a cyclic phosphodiesterase (CPDase) motif (Nasr and Filipowicz, 2000; Mazumder et al., 2002; Myllykoski et al., 2013), human USB1 lacks the CPDase activity and thus generates a cP as a final form. In contrast, yeast Usb1 retains the CPDase activity (Didychuk et al., 2017), generating 3′-P end of U6 snRNA (Lund and Dahlberg, 1992). It will be intriguing to address how and why the difference arose.

rRNA maturation requires a cP-forming endoribonuclease, Lethal in the absence of Ssd1 (Las1 in yeast; Las1L in human) (Castle et al., 2010; Schillewaert et al., 2012). In yeast, rRNA maturation starts from processing of a nascent 37S rRNA precursor into shorter precursors, including 27S rRNA (Henras et al., 2015; Gerstberger et al., 2017). The 27S rRNA is further cleaved by Las1 between 5.8S and 25S rRNA sequences, generating 7S rRNA as a 5′-cleavage product with a cP (Gasse et al., 2015; Pillon et al., 2017). The cleavage is catalyzed by an N-terminal α-helical ‘higher eukaryotes and prokaryotes nucleotide-binding’ (HEPN) domain of Las1, which has been defined as a conserved RΦxxxH catalytic motif (Φ: N, D, or H) (Anantharaman et al., 2013; Pillon et al., 2017). During further processing of 7S rRNA into mature 5.8S rRNA, a cP end of 7S rRNA is removed and processed by unknown mechanisms; therefore, cP is absent in mature rRNAs.

cP is also formed in a mRNA splicing event that plays a crucial role in activating the unfolded protein response (UPR) pathway upon endoplasmic reticulum (ER) stress. Inositol-requiring enzyme 1 (Ire1), a cP-forming endonuclease, is associated with the ER membrane with its C-terminal domain exposed to the cytosol (Urano et al., 2000; Zhang and Kaufman, 2004). While an interaction with ‘binding immunoglobulin protein’ (BiP), an ER chaperon protein, retains Ire1 as an inactive monomer under normal conditions, ER stress releases BiP, allowing Ire1 to form a homodimer that harbors an active nuclease domain. The activated Ire1 is involved in splicing of *HAC1* mRNA (in yeast; *XBP1* mRNA in human) by cleaving both 5′- and 3′-splice sites, in which a cP is formed at the conserved 3′-terminal G of 5′-cleavage products (Sidrauski and Walter, 1997; Gonzalez et al., 1999; Shinya et al., 2011) (Figure 3D). From the spliced, mature form of *HAC1* mRNA, a basic-region leucine-zipper transcription factor HAC1 is expressed, eventually promoting the transcription of its target genes containing UPR-responsive elements (Sidrauski and Walter, 1997; Urano et al., 2000; Zhang and Kaufman, 2004).

cP-forming endoribonucleases are further found in colicins, toxic proteins that are encoded in plasmid DNAs in some *E. coli* strains to invade and kill other bacteria (Cascales et al., 2007). Among over 20 colicins identified thus far, colicin E5 and D have been shown to cleave the anticodon-loop of tRNAs and form a cP (Ogawa et al., 1999; Ogawa et al., 2006) (Figure 3E). While endoribonuclease activity of those colicins is masked by immunity proteins in host *E. coli*, colicin E5 invades other bacteria and cleaves tRNA^TyrGUA^, tRNA^HisGUG^, tRNA^AsnGUU^, and tRNA^AspGUC^ between G at nucleotide position 34 (G_34_) and U_35_ (Ogawa et al., 1999, 2006), and colicin D cleaves all four isoacceptors of tRNA^Arg^ between A_38_ and G_39_/C_39_ (Tomita et al., 2000), contributing to bacterial lethality.

The *E. coli* genome also encodes cP-forming endoribonucleases involved in toxin-antitoxin (TA) systems. TA systems involve bacterial stress responses, often considered “suicidal programs,” comprising a stable toxin and an unstable antitoxin that neutralizes the cognate toxin in cells (Unterholzner et al., 2013). In the well-studied MazEF system (Figure 3F), toxic endoribonuclease MazF is neutralized by antitoxin MazE under normal conditions, but various stresses, such as nutrient limitation, DNA damage, and antibiotic exposure, degrade MazE and thereby release MazF (Jensen and Gerdes, 1995; Yarmolinsky, 1995; Engelberg-Kulka and Glaser, 1999) which cleaves whole cellular mRNAs to prevent further protein production (Zhang et al., 2003). MazF cleaves the 5′-side of an ACA motif within mRNAs, and forms a cP (Zhang et al., 2003, 2005a; Vesper et al., 2011). Recent reports showed MazF-catalyzed cleavage of 16S and 23S rRNAs, and some tRNAs such as tRNA^LysUUU^ (Vesper et al., 2011; Moll and Engelberg-Kulka, 2012; Schifano et al., 2013; Schifano et al., 2014, 2016; Mets et al., 2017), indicating that MazF is a critical suicide factor causing perturbation of the whole cellular transcriptome. The ChpBIK system, another TA system, also uses a cP-forming enzyme as a toxin. When released from antitoxin ChpBI under stress conditions, toxic endoribonuclease ChpBK cleaves mRNAs at the 5′- or 3′-side of A in an ACY sequence motif to prevent further protein production (Christensen et al., 2003; Zhang et al., 2005b). The 5′-cleavage products contain.

The genome of some *E. coli* isolates possesses a *prr* locus, encoding PrrC endonuclease (also known as anticodon nuclease: ACNase), which is considered to be another bacterial suicide program (Kaufmann, 2000). PrrC activity is usually silenced by interaction with a masking protein, but, upon T4 phage infection, it forms an ACNase complex and cleaves tRNA^LysUUU^ between U_33_ and U_34_, which serve as a host defense to inhibit translation of T4 proteins (Amitsur et al., 1987; Morad et al., 1993). The 5′-tRNA^LysUUU^ half resulting from the PrrC cleavage harbors a cP.

cP-forming cytotoxic endoribonucleases are also present in eukaryotes. Zymocin and PaT are toxin complexes secreted by the yeasts *Kluyveromyces lactis* and *Pichia acaciae*, respectively, to inhibit the growth of other yeasts (Lu et al., 2005, 2008; Klassen et al., 2008) (Figure 3E). Zymocin is composed of the three subunits; two of them assist target cell binding and invasion, while the remaining γ-subunit cleaves tRNAs in targeted yeasts (Stark and Boyd, 1986). The γ-subunit of zymocin recognizes a 5-methoxycarbonylmethyl-2-thiouridine (mcm^5^s^2^U), a specific modified RNA nucleotide present at np 34 of tRNA^GluUUC^, tRNA^LysUUU^, and tRNA^GlnUUG^, and cleaves between U_34_ and U_35_ of those tRNAs (Lu et al., 2005), leaving a cP at the ribose of mcm^5^s^2^U in the cleavage products. PaT is a heterodimer composed of PaOrf1, a cell invasion-assisting subunit, and PaOrf2, an endonuclease subunit (McCracken et al., 1994; Klassen et al., 2004). PaOrf2 recognizes 5-methoxycarbonylmethyl uridine (mcm^5^U) and cleaves between U_34_ and U_35_ of tRNA^GlnUUG^, leaving a cP at the ribose of mcm^5^U in the cleavage product (Klassen et al., 2008; Chakravarty et al., 2014).

cP-forming endoribonucleases are further found in viruses. DNA topoisomerase, encoded in vaccinia virus, belongs to the type IB family of eukaryotic DNA topoisomerases and uniquely harbors endoribonucleolytic activity, which forms a cP end at the cleaved RNAs (Sekiguchi and Shuman, 1997; Shuman, 1998). Analogous to yeast topoisomerase I, which can remove single ribonucleotides in DNA duplexes (Kim et al., 2011), topoisomerase’s RNA cleavage activity might be involved in maintaining genome integrity during DNA replication. Replicative nidoviral uridylate-specific endoribonuclease (NendoU), encoded in nidovirus, is also a cP-forming endoribonuclease (Ivanov et al., 2004). While the functional role of the endoribonuclease activity in virus infection and replication is not fully understood, NendoU preferentially targets dsRNA and cleaves the 5′-side of uridine in G–U or G–U–U sequence to generate cP-RNAs (Ivanov et al., 2004).

### Ribozymes

Ribozymes are another cP-yielding biocatalyst. Among several distinct classes of ribozymes, a class of small, self-cleaving ribozymes is known to generate cPs (Scott and Klug, 1996; Doherty and Doudna, 2001; Serganov and Patel, 2007). Small self-cleaving ribozymes are widely found in bacterial, plant, and mammalian genomes, and are involved in gene controls and expressions (Shih and Been, 2002; Serganov and Patel, 2007). Out of 11 identified ribozymes in this class, 10 have been shown to form cP ends as a result of their cleavage of RNAs (Saville and Collins, 1990; Scott and Klug, 1996; Winkler et al., 2004; Salehi-Ashtiani et al., 2006; Roth et al., 2014; Harris et al., 2015; Li et al., 2015; Weinberg et al., 2015). In the case of the hepatitis delta virus (HDV) ribozyme, the 85-nt minimal self-cleavage domain cleaves between U_−1_ and G_1_ (Shih and Been, 2002; Puerta-Fernandez et al., 2003). While C_75_ is suggested to act as a general acid catalyst by donating a proton from its N3 in the pyrimidine ring to a leaving group, several different molecules have been proposed as potential base catalysts: water or hydroxide from the solvent, water molecules coordinated to the Mg^2+^, or 2′-OH of G_27_ positioned closely adjacent to the catalytic site (Ward et al., 2014).

### Enzymes That Act Directly on the 3′-End of RNAs

There are two protein catalysts that have been reported to form a cP by a distinct molecular mechanism from transesterification during RNA cleavage. As described above, RtcA can catalyze *de novo* cP formation by directly acting on the 3′-end of RNAs (Genschik et al., 1997, 1998; Billy et al., 2000; Filipowicz, 2016). Although endogenous RNA target of RtcA is unknown, in the *E. coli* genome, *rtcA* and an RNA ligase *rtcB* form an *rtcBA* operon, which is implicated in RNA repair pathway (Das and Shuman, 2013a; Burroughs and Aravind, 2016). Archaeal thermophilic RNA ligase from *Methanobacterium thermoautotrophicum*, MthRnl, is the other enzyme which can also catalyze *de novo* cP formation (Zhelkovsky and McReynolds, 2014; Yoshinari et al., 2017). While MthRnl can ligate 5′-P and 3′-OH ends of RNAs (Torchia et al., 2008), when substrate RNAs contain a 3′-P, MthRnl coverts it to a 3′-cP by a similar mechanism with RtcA (Zhelkovsky and McReynolds, 2014). In addition, MthRnl possesses the 3′-deadenylation activity which can remove a 3′-terminal adenosine with an OH end and form a cP (Yoshinari et al., 2017). Endogenous RNA target of MthRnl is unknown.

## Biological Significance of cP Formation and cP-Rna Expression

What is the significance of cP formation in RNAs? It has been shown that cP formation in U6 snRNA regulates RNA interaction with protein factors. While nascent U6 snRNA containing 3′-OH end is bound by La protein (Maraia and Intine, 2002; Maraia and Bayfield, 2006), cP formation of mature U6 snRNA promotes interaction with Lsm2–8 complexes (Khusial et al., 2005; Licht et al., 2008) (Figure 3C). The affinity of the cP-containing RNA to Lsm2–8 is higher than 3′-OH-containing RNA, and the interaction of La/3′-OH and Lsm2–8/cP is mutually exclusive: even when both La and Lsm2–8 exist in the reaction solution, RNA with 3′-OH or with cP only binds to La or Lsm2–8, respectively (Licht et al., 2008). cP formation is, therefore, a critical factor for forming functional spliceosome complexes with Lsm2–8 (Didychuk et al., 2018). Although this is the only proven example of the cP-regulated formation of RNA-protein complex, cP formation in other cP-RNAs may modulate RNA interaction with protein factors.

RNA ligation reaction can depend on a cP in a substrate RNA. In tRNA splicing, Sen2-mediated cleavage forms a 3′-terminal cP in 5′-exons, which is then ligated to the 5′-OH end of 3′-exons by tRNA ligase (Popow et al., 2012; Yoshihisa, 2014) (Figure 3A). In *Arabidopsis thaliana*, the tRNA ligase AtRNL is able to ligate cP ends to 3′-exons but cannot use 3′-P ends as its ligation substrate (Schutz et al., 2010; Tanaka et al., 2011a). This cP-specific ligation activity was also observed in wheat germ extract (Konarska et al., 1982). In this plant ligation process, cP ends of 5′-exons are first converted to 2′-P and 3′-OH. 5′-OH ends of 3′-exons are phosphorylated, followed by ligation to 3′-OH of 5′-exons (Popow et al., 2012; Yoshihisa, 2014). Other organisms employ distinct molecular mechanisms in ligation of cP-containing 5′-exons to 3′-exons in tRNA splicing (Popow et al., 2012; Yoshihisa, 2014). In humans, RtcB was identified as a tRNA ligase (Popow et al., 2011). Experiments using lysates and RtcB immunoprecipitates from HeLa cells suggest that human RtcB prefers cP and 5′-OH for ligation (Filipowicz et al., 1983; Popow et al., 2011). However, whether the substrate specificity extends to 3′-P and 5′-OH containing RNA still awaits analysis using a recombinant human tRNA ligase complex. In mammals, RtcB is involved in splicing of *XBP1* mRNA in the UPR pathway (Filipowicz, 2014; Jurkin et al., 2014; Lu et al., 2014) (Figure 3D). *E. coli* RtcB is also able to ligate cP and 5′-OH, as well as 3′-P and 5′-OH (Tanaka and Shuman, 2011; Tanaka et al., 2011b). In the ligation, cP is first converted to 3′-P, then ligated to 5′-OH (Tanaka et al., 2011a). *E. coli* RtcB can catalyze the re-ligation of 16S rRNA at the site cleaved by stress-induced MazF activity, which generates full-length 16S rRNA and contributes to restoration from the stress conditions (Temmel et al., 2017).

Besides influencing interaction and activity of proteins, cP formation may play a role in stabilizing RNA molecules by protecting them from degradation. Ehrlich exoribonuclease extracted from Ehrlich ascites cells and various mouse tissues, later defined as exoribonuclease II, was shown to degrade single-stranded RNAs with 3′-OH ends more rapidly than those with cP and 3′-P ends (Sporn et al., 1969), suggesting that cP formation is advantageous for RNA molecules to exist stably in cells. In contrast, because RNAs with 3′-P ends are more rapidly degraded by the exosome complex exoribonuclease, Rrp44, than those with 3′-OH ends (Zinder et al., 2016), cP formation could also negatively impact the stability of cP-RNAs. Thus, a cP structure might be able to regulate RNA stability in both directions by affecting degradation activity of nucleases or modulating RNA-protein interactions. Further study is required to shed more light on the potential function of cP formation in RNA stability.

The above described advantages of cP formation may, in turn, suggest the biological significance of cellular cP-RNA expression. While the functional significance of U6 snRNA, which belongs to cP-RNAs, or tRNAs and rRNAs, whose biogenesis is intermediated by cP-RNAs, have been apparent for a long time, previously uncharacterized cP-RNAs are now being demonstrated as functional molecules which play important roles in various biological processes. Representative examples of such functional cP-RNAs include the 5′-tRNA half molecules. In mammalian cells, various stress stimuli trigger ANG-mediated tRNA cleavage to produce functional tRNA halves, termed tRNA-derived stress-induced RNAs (tiRNAs) (Fu et al., 2009; Yamasaki et al., 2009) (Figure 3B). 5′-tiRNAs, corresponding to 5′-tRNA halves, have been shown as functional molecules that can promote formation of stress granules and regulate translation via YB-1 protein-involved pathway (Emara et al., 2010; Ivanov et al., 2011, 2014; Lyons et al., 2016).

ANG-mediated tRNA cleavage is also promoted by sex hormone signaling pathways in hormone dependent breast and prostate cancer cells, generating a distinct class of tRNA halves termed sex hormone-dependent tRNA-derived RNAs (SHOT-RNAs) (Honda et al., 2015; Honda and Kirino, 2016) (Figure 3B). 5′-SHOT-RNAs, belonging to cP-RNAs, promote cell proliferation. The expression levels of SHOT-RNAs in tissues and serum of prostate cancer patients have been shown to be associated with pathological and prognostic parameters, suggesting the use of SHOT-RNAs as potential diagnostic biomarkers (Zhao et al., 2018). In terms of diseases, many different *ANG* gene mutations have been identified in patients with amyotrophic lateral sclerosis (ALS) and Parkinson’s disease (Tello-Montoliu et al., 2006; Gao and Xu, 2008), implying that ANG-catalyzed production of tRNA halves could be involved in the pathogenesis of these neurodegenerative disorders (Thiyagarajan et al., 2012). Indeed, accumulation of tRNA halves contributes to the pathogenesis of a syndromic form of intellectual disability and Dubowitz-like syndrome (Blanco et al., 2014).

cP-RNAs can also function as direct precursors for shorter functional RNAs. In *B. mori* germ cells, some abundant species of Piwi-interacting RNAs (piRNAs), a germline-specific class of small regulatory RNAs, are produced directly from cP-containing 5′-tRNA halves (Honda et al., 2017) (Figure 3G). Although many microRNAs (miRNAs) are derived from tRNAs (Shigematsu and Kirino, 2015; Telonis et al., 2015), whether the tRNA-derived miRNAs are also generated from cP-containing tRNA halves has not been examined yet. Further research may reveal more evidence of cP-RNA uses as direct precursors for functional RNAs.

## Specific Sequencing and Quantification of cP-Rnas

**FIGURE 4:**
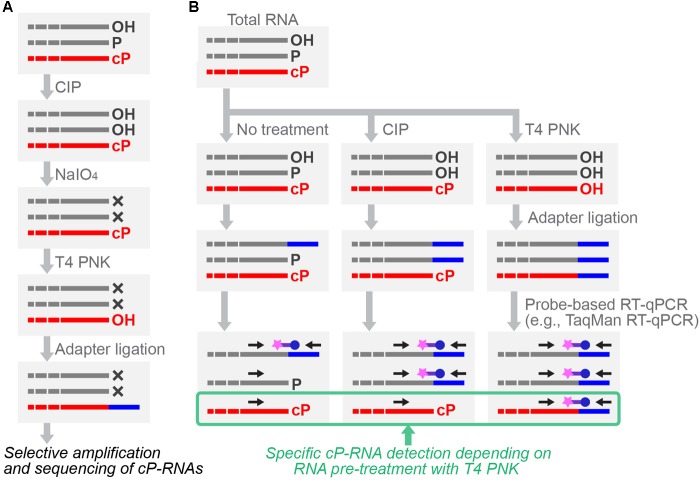
Schematic representation of cP-RNA-seq **(A)** and probe-based RT-qPCR **(B)** for specific cP-RNA analyses.

To further expand cP-RNA research, it is imperative to capture cP-RNA expression profiles accurately, which is not possible using standard RNA-seq methods. Specific cP-RNA sequencing can be achieved by cP-RNA-seq (Honda et al., 2015, 2016) which takes advantage of distinct properties of two well-used enzymes, T4 polynucleotide kinase (T4 PNK) and a phosphatase such as calf intestinal phosphatase (CIP). T4 PNK has 3′-terminal phosphatase activity that removes both a P and cP from the 3′-end of RNAs (Amitsur et al., 1987; Das and Shuman, 2013b), whereas CIP removes only a P but not a cP. In cP-RNA-seq, RNAs are first treated with CIP to remove a P, followed by periodate oxidization. Because the oxidation cleaves the 3′-end of all RNAs other than cP-RNAs, subsequent cP removal, adapter ligation, and cDNA amplification steps are exclusively applied to cP-RNAs, leading to selective amplification and sequencing of cP-RNAs (Honda et al., 2015, 2016) (Figure 4A). cP-RNA-seq only requires commercially available enzymes and reagents, which is an advantage of the method. As a limitation of the method, RNAs lacking a 2′,3′-diol structure of ribose, such as plant miRNAs and animal piRNAs that contain 2′-*O*-methyl ribose modification (Yang et al., 2006; Kirino and Mourelatos, 2007; Ohara et al., 2007), can also be amplified despite the absence of a cP, because those RNAs would be resistant to periodate oxidation. This point should always be remembered especially when 20–30-nt small RNAs are used for the method. Thus far, cP-RNA-seq has been applied only to the two cell lines, human BT-474 breast cancer cells and *B. mori* BmN4 cells (Honda et al., 2015, 2017). Although high mapping ratio of the obtained reads to tRNA sequences showed the specificity and credibility of the method, both of the studies narrowly focused on short RNA fraction containing tRNA haves. Further application of the method to broader RNA populations will enable more global identification of cP-RNA species.

As an alternative method, *Arabidopsis* tRNA ligase AtRNL can be used for specific cP-RNA sequencing (Schutz et al., 2010). Because its ligation activity is specific to a cP but not to a 3′-P and 3′-OH, AtRNL selectively ligates a 3′-adapter to cP-RNAs among all RNA species. After the ligation, for efficient reverse transcription, a 2′-P formed at the substrate–adapter junction should be removed by 2′-phosphotransferase treatment. Therefore, two specific recombinant proteins, AtRNL and *Saccharomyces cerevisiae* 2′-phosphotransferase Tpt1, were purified and used in the method (Schutz et al., 2010). Application of the method to human brain total RNA identified numerous reads of cP-RNAs containing U6 snRNA. The 3′-ends of ∼90% of the U6 snRNA reads were identified as a consistent, mature form, validating the specificity and credibility of the method. Considering the ligation activity for cP-RNAs, RtcB can also be used for cP-RNA sequencing (Donovan et al., 2017). Because RtcB can ligate 3′-P ends, as well as cP ends, a phosphatase treatment to remove 3′-P prior to RtcB-mediated 3′-adaptor ligation would be required for specific capture of cP-RNAs.

After cP-RNA sequencing, amplification and quantification of the representative identified cP-RNA species are necessary to validate their expression and analyze whether a cP end is the major 3′-end form of the identified sequences. Standard RT-qPCR, amplifying internal sequences of targeted RNAs, is inappropriate for specific amplification of cP-RNAs because it cannot distinguish between cP-RNAs and RNAs with other terminal states. To specifically analyze cP-RNAs, RNAs treated with T4 PNK or CIP can be subjected to 3′-adapter ligation, followed by TaqMan RT-qPCR targeting 3′-adapter-RNA ligation products (Honda and Kirino, 2015; Honda et al., 2015, 2017; Shigematsu et al., 2018; Zhao et al., 2018) (Figure 4B). The dependency of amplification signals on RNA treatment with T4 PNK, but not with CIP, allows researchers to confirm that the detected signals are derived from cP-RNAs because they should be ligated to a 3′-adapter only after cP removal by T4 PNK treatment. As an alternative method for analyzing cP ends, T4 PNK- or CIP-treated RNAs can be subjected to a poly (A) polymerase reaction which is able to add poly (A) tails to 3′-OH ends, but not to cP ends (Zaug et al., 1996). Moreover, northern blot can be used to observe slight differences in band mobility between cP-RNAs and RNAs with other terminal states (Honda et al., 2015, 2016).

## Future Perspectives

Despite the findings described in this review, current information regarding cellular expression profiles of cP-RNAs is very limited and fragmented. Although increasing accumulation of RNA-seq data has accelerated the comparative analyses of transcriptomes and, therefore, been critical to identifying significant RNA species in biological phenomena and diseases, the “invisibility” of cP-RNA expression in RNA-seq data makes cP-RNA research be still at an initial stage. The immediate future focus should be on capturing the comprehensive repertoire of cP-RNAs expressed in different tissues and cells by using the above described specific sequencing methods. Given that cP-RNAs are expressed as functional molecules, capturing the entire cP-RNA repertoire would broaden the catalog of functional non-coding RNAs and could reveal significant biological events that have been eluding standard RNA-seq. Besides cP-RNA expression, molecular mechanisms behind cP-RNA biogenesis and function still remain elusive. Presumably, not all cP-RNA-producing enzymes have been identified and characterized to date. Because determining cP-RNA-generating enzymes only by their amino-acid sequences and protein motifs is impossible, discovering novel cP-RNA-generating enzymes will rely on detailed structural and biochemical characterizations of each enzyme. Given the already-proven biological roles of cP formation and cP-RNA expression, it is not surprising that cP-RNAs are involved in a wide range of biological processes. Considering the “hidden” nature of cP-RNAs in conventional RNA-seq data, further research efforts to characterize cP-RNAs would likely clarify substantially greater biological significance of cP-RNAs, which will advance our understanding of the expanding realm of non-coding RNA molecules.

## Author Contributions

MS and YK conceptualized the theme and wrote the review with substantial help by TK in compiling reference papers. All authors reviewed and approved the final manuscript.

## Conflict of Interest Statement

The authors declare that the research was conducted in the absence of any commercial or financial relationships that could be construed as a potential conflict of interest.

## References

[B1] Abelson J. Trotta C. R. Li H. (1998). tRNA splicing. *J. Biol. Chem.* 273 12685–12688. 10.1074/jbc.273.21.12685 9582290

[B2] Acharya K. R. Shapiro R. Allen S. C. Riordan J. F. Vallee B. L. (1994). Crystal structure of human angiogenin reveals the structural basis for its functional divergence from ribonuclease. *Proc. Natl. Acad. Sci. U.S.A.* 91 2915–2919. 10.1073/pnas.91.8.2915 8159679 PMC43485

[B3] Amitsur M. Levitz R. Kaufmann G. (1987). Bacteriophage T4 anticodon nuclease, polynucleotide kinase and RNA ligase reprocess the host lysine tRNA. *EMBO J.* 6 2499–2503. 10.1002/j.1460-2075.1987.tb02532.x 2444436 PMC553660

[B4] Anantharaman V. Makarova K. S. Burroughs A. M. Koonin E. V. Aravind L. (2013). Comprehensive analysis of the HEPN superfamily: identification of novel roles in intra-genomic conflicts, defense, pathogenesis and RNA processing. *Biol. Direct* 8:15. 10.1186/1745-6150-8-151745-6150-8-15 23768067 PMC3710099

[B5] Bartlett G. J. Porter C. T. Borkakoti N. Thornton J. M. (2002). Analysis of catalytic residues in enzyme active sites. *J. Mol. Biol.* 324 105–121. 10.1016/S0022-2836(02)01036-7 12421562

[B6] Bierhals T. Korenke G. C. Uyanik G. Kutsche K. (2013). Pontocerebellar hypoplasia type 2 and TSEN2: review of the literature and two novel mutations. *Eur. J. Med. Genet.* 56 325–330. 10.1016/j.ejmg.2013.03.009S1769-7212(13)00081-5 23562994

[B7] Billy E. Wegierski T. Nasr F. Filipowicz W. (2000). Rcl1p, the yeast protein similar to the RNA 3′-phosphate cyclase, associates with U3 snoRNP and is required for 18S rRNA biogenesis. *EMBO J.* 19 2115–2126. 10.1093/emboj/19.9.2115 10790377 PMC305690

[B8] Blanco S. Dietmann S. Flores J. V. Hussain S. Kutter C. Humphreys P. (2014). Aberrant methylation of tRNAs links cellular stress to neuro-developmental disorders. *EMBO J.* 33 2020–2039. 10.15252/embj.201489282embj.201489282 25063673 PMC4195770

[B9] Budde B. S. Namavar Y. Barth P. G. Poll-The B. T. Nurnberg G. Becker C. (2008). tRNA splicing endonuclease mutations cause pontocerebellar hypoplasia. *Nat. Genet.* 40 1113–1118. 10.1038/ng.204ng.204 18711368

[B10] Burroughs A. M. Aravind L. (2016). RNA damage in biological conflicts and the diversity of responding RNA repair systems. *Nucleic Acids Res.* 44 8525–8555. 10.1093/nar/gkw722 27536007 PMC5062991

[B11] Cascales E. Buchanan S. K. Duche D. Kleanthous C. Lloubes R. Postle K. (2007). Colicin biology. *Microbiol. Mol. Biol. Rev.* 71 158–229. 10.1128/MMBR.00036-06 17347522 PMC1847374

[B12] Castle C. D. Cassimere E. K. Lee J. Denicourt C. (2010). Las1L is a nucleolar protein required for cell proliferation and ribosome biogenesis. *Mol. Cell Biol.* 30 4404–4414. 10.1128/MCB.00358-10 20647540 PMC2937536

[B13] Chakravarty A. K. Smith P. Jalan R. Shuman S. (2014). Structure, mechanism, and specificity of a eukaryal tRNA restriction enzyme involved in self-nonself discrimination. *Cell Rep.* 7 339–347. 10.1016/j.celrep.2014.03.034 24726365 PMC4121121

[B14] Chan P. P. Lowe T. M. (2016). GtRNAdb 2.0: an expanded database of transfer RNA genes identified in complete and draft genomes. *Nucleic Acids Res.* 44 D184–D189. 10.1093/nar/gkv1309 26673694 PMC4702915

[B15] Cho S. Zhang J. (2006). Ancient expansion of the ribonuclease A superfamily revealed by genomic analysis of placental and marsupial mammals. *Gene* 373 116–125. 10.1016/j.gene.2006.01.018 16530354

[B16] Christensen S. K. Pedersen K. Hansen F. G. Gerdes K. (2003). Toxin-antitoxin loci as stress-response-elements: ChpAK/MazF and ChpBK cleave translated RNAs and are counteracted by tmRNA. *J. Mol. Biol.* 332 809–819. 10.1016/S0022-2836(03)00922-7 12972253

[B17] Cuchillo C. M. Nogues M. V. Raines R. T. (2011). Bovine pancreatic ribonuclease: fifty years of the first enzymatic reaction mechanism. *Biochemistry* 50 7835–7841. 10.1021/bi201075b 21838247 PMC3172371

[B18] Cuchillo C. M. Vilanova M. Nogues V. (1997). “”Pancreatic ribonucleases,” in *Ribonucleases: Structures and Functions*, eds Alessio G. D. Riordan J. F. (Cambridge, MA: Academic Press),272–297.

[B19] Das U. Shuman S. (2013a). 2′-Phosphate cyclase activity of RtcA: a potential rationale for the operon organization of RtcA with an RNA repair ligase RtcB in *Escherichia coli* and other bacterial taxa. *RNA* 19 1355–1362. 10.1261/rna.039917.113 23945037 PMC3854526

[B20] Das U. Shuman S. (2013b). Mechanism of RNA 2′,3′-cyclic phosphate end healing by T4 polynucleotide kinase-phosphatase. *Nucleic Acids Res.* 41 355–365. 10.1093/nar/gks977 23118482 PMC3592404

[B21] Didychuk A. L. Butcher S. E. Brow D. A. (2018). The life of U6 small nuclear RNA, from cradle to grave. *RNA* 24 437–460. 10.1261/rna.065136.117 29367453 PMC5855946

[B22] Didychuk A. L. Montemayor E. J. Carrocci T. J. DeLaitsch A. T. Lucarelli S. E. Westler W. M. (2017). Usb1 controls U6 snRNP assembly through evolutionarily divergent cyclic phosphodiesterase activities. *Nat. Commun.* 8:497. 10.1038/s41467-017-00484-w 28887445 PMC5591277

[B23] Doherty E. A. Doudna J. A. (2001). Ribozyme structures and mechanisms. *Annu. Rev. Biophys. Biomol. Struct.* 30 457–475. 10.1146/annurev.biophys.30.1.457 11441810

[B24] Donovan J. Rath S. Kolet-Mandrikov D. Korennykh A. (2017). Rapid RNase L-driven arrest of protein synthesis in the dsRNA response without degradation of translation machinery. *RNA* 23 1660–1671. 10.1261/rna.062000.117 28808124 PMC5648034

[B25] Dyer K. D. Rosenberg H. F. (2006). The RNase a superfamily: generation of diversity and innate host defense. *Mol. Divers.* 10 585–597. 10.1007/s11030-006-9028-2 16969722

[B26] Emara M. M. Ivanov P. Hickman T. Dawra N. Tisdale S. Kedersha N. (2010). Angiogenin-induced tRNA-derived stress-induced RNAs promote stress-induced stress granule assembly. *J. Biol. Chem.* 285 10959–10968. 10.1074/jbc.M109.077560 20129916 PMC2856301

[B27] Engelberg-Kulka H. Glaser G. (1999). Addiction modules and programmed cell death and antideath in bacterial cultures. *Annu. Rev. Microbiol.* 53 43–70. 10.1146/annurev.micro.53.1.43 10547685

[B28] Fabian H. Mantsch H. H. (1995). Ribonuclease A revisited: infrared spectroscopic evidence for lack of native-like secondary structures in the thermally denatured state. *Biochemistry* 34 13651–13655. 10.1021/bi00041a046 7577955

[B29] Fett J. W. Strydom D. J. Lobb R. R. Alderman E. M. Bethune J. L. Riordan J. F. (1985). Isolation and characterization of angiogenin, an angiogenic protein from human carcinoma cells. *Biochemistry* 24 5480–5486. 10.1021/bi00341a030 4074709

[B30] Filipowicz W. (2014). Making ends meet: a role of RNA ligase RTCB in unfolded protein response. *EMBO J.* 33 2887–2889. 10.15252/embj.201490425 25404664 PMC4282637

[B31] Filipowicz W. (2016). RNA 3′-terminal phosphate cyclases and cyclase-like proteins. *Postepy Biochem.* 62 327–334. 28132487

[B32] Filipowicz W. Konarska M. Gross H. J. Shatkin A. J. (1983). RNA 3′-terminal phosphate cyclase activity and RNA ligation in HeLa cell extract. *Nucleic Acids Res.* 11 1405–1418. 10.1093/nar/11.5.1405 6828385 PMC325805

[B33] Fu H. Feng J. Liu Q. Sun F. Tie Y. Zhu J. (2009). Stress induces tRNA cleavage by angiogenin in mammalian cells. *FEBS Lett.* 583 437–442. 10.1016/j.febslet.2008.12.043 19114040

[B34] Gao X. Xu Z. (2008). Mechanisms of action of angiogenin. *Acta Biochim. Biophys. Sin.* 40 619–624. 10.1111/j.1745-7270.2008.00442.x 18604453

[B35] Gasse L. Flemming D. Hurt E. (2015). Coordinated ribosomal ITS2 RNA processing by the las1 complex integrating endonuclease, polynucleotide kinase, and exonuclease activities. *Mol. Cell* 60 808–815. 10.1016/j.molcel.2015.10.021 26638174

[B36] Genschik P. Billy E. Swianiewicz M. Filipowicz W. (1997). The human RNA 3′-terminal phosphate cyclase is a member of a new family of proteins conserved in Eucarya, Bacteria and Archaea. *EMBO J.* 16 2955–2967. 10.1093/emboj/16.10.2955 9184239 PMC1169903

[B37] Genschik P. Drabikowski K. Filipowicz W. (1998). Characterization of the *Escherichia coli* RNA 3′-terminal phosphate cyclase and its sigma54-regulated operon. *J. Biol. Chem.* 273 25516–25526. 10.1074/jbc.273.39.25516 9738023

[B38] Gerstberger S. Meyer C. Benjamin-Hong S. Rodriguez J. Briskin D. Bognanni C. (2017). The conserved RNA exonuclease Rexo5 is required for 3′ end maturation of 28S rRNA, 5S rRNA, and snoRNAs. *Cell Rep.* 21 758–772. 10.1016/j.celrep.2017.09.067 29045842 PMC5662206

[B39] Gonzalez T. N. Sidrauski C. Dorfler S. Walter P. (1999). Mechanism of non-spliceosomal mRNA splicing in the unfolded protein response pathway. *EMBO J.* 18 3119–3132. 10.1093/emboj/18.11.3119 10357823 PMC1171393

[B40] Harper J. W. Vallee B. L. (1989). A covalent angiogenin/ribonuclease hybrid with a fourth disulfide bond generated by regional mutagenesis. *Biochemistry* 28 1875–1884. 10.1021/bi00430a067 2719939

[B41] Harris K. A. Lunse C. E. Li S. Brewer K. I. Breaker R. R. (2015). Biochemical analysis of pistol self-cleaving ribozymes. *RNA* 21 1852–1858. 10.1261/rna.052514.115 26385507 PMC4604425

[B42] Henras A. K. Plisson-Chastang C. O’Donohue M. F. Chakraborty A. Gleizes P. E. (2015). An overview of pre-ribosomal RNA processing in eukaryotes. *Wiley Interdiscip. Rev. RNA* 6 225–242. 10.1002/wrna.1269 25346433 PMC4361047

[B43] Hilcenko C. Simpson P. J. Finch A. J. Bowler F. R. Churcher M. J. Jin L. (2013). Aberrant 3′ oligoadenylation of spliceosomal U6 small nuclear RNA in poikiloderma with neutropenia. *Blood* 121 1028–1038. 10.1182/blood-2012-10-461491 23190533

[B44] Holloway D. E. Chavali G. B. Hares M. C. Baker M. D. Subbarao G. V. Shapiro R. (2004). Crystallographic studies on structural features that determine the enzymatic specificity and potency of human angiogenin: Thr44, Thr80, and residues 38-41. *Biochemistry* 43 1230–1241. 10.1021/bi035654+ 14756559

[B45] Honda S. Kawamura T. Loher P. Morichika K. Rigoutsos I. Kirino Y. (2017). The biogenesis pathway of tRNA-derived piRNAs in Bombyx germ cells. *Nucleic Acids Res.* 45 9108–9120. 10.1093/nar/gkx537 28645172 PMC5587776

[B46] Honda S. Kirino Y. (2015). Dumbbell-PCR: a method to quantify specific small RNA variants with a single nucleotide resolution at terminal sequences. *Nucleic Acids Res.* 43:e77. 10.1093/nar/gkv218 25779041 PMC4499115

[B47] Honda S. Kirino Y. (2016). SHOT-RNAs: a novel class of tRNA-derived functional RNAs expressed in hormone-dependent cancers. *Mol. Cell. Oncol.* 3:e1079672. 10.1080/23723556.2015.1079672 27308603 PMC4905382

[B48] Honda S. Loher P. Shigematsu M. Palazzo J. P. Suzuki R. Imoto I. (2015). Sex hormone-dependent tRNA halves enhance cell proliferation in breast and prostate cancers. *Proc. Natl. Acad. Sci. U.S.A.* 112 E3816–E3825. 10.1073/pnas.1510077112 26124144 PMC4517238

[B49] Honda S. Morichika K. Kirino Y. (2016). Selective amplification and sequencing of cyclic phosphate-containing RNAs by the cP-RNA-seq method. *Nat. Protoc.* 11 476–489. 10.1038/nprot.2016.025 26866791 PMC4852555

[B50] Hopper A. K. Phizicky E. M. (2003). tRNA transfers to the limelight. *Genes Dev.* 17 162–180. 10.1101/gad.1049103 12533506

[B51] Inoue-Ito S. Yajima S. Fushinobu S. Nakamura S. Ogawa T. Hidaka M. (2012). Identification of the catalytic residues of sequence-specific and histidine-free ribonuclease colicin E5. *J. Biochem.* 152 365–372. 10.1093/jb/mvs077 22815490

[B52] Irie M. (1997). “”RNase T1/RNase T2 family RNases,” in *Ribonucleases: Structures and Functions*, eds Alessio G. D. Riordan J. F. (Cambridge, MA: Academic Press), 101–124.

[B53] Ivanov K. A. Hertzig T. Rozanov M. Bayer S. Thiel V. Gorbalenya A. E. (2004). Major genetic marker of nidoviruses encodes a replicative endoribonuclease. *Proc. Natl. Acad. Sci. U.S.A.* 101 12694–12699. 10.1073/pnas.0403127101 15304651 PMC514660

[B54] Ivanov P. Emara M. M. Villén J. Gygi S. P. Anderson P. (2011). Angiogenin-induced tRNA fragments inhibit translation initiation. *Mol. Cell* 43 613–623. 10.1016/j.molcel.2011.06.022 21855800 PMC3160621

[B55] Ivanov P. O’Day E. Emara M. M. Wagner G. Lieberman J. Anderson P. (2014). G-quadruplex structures contribute to the neuroprotective effects of angiogenin-induced tRNA fragments. *Proc. Natl. Acad. Sci. U.S.A.* 111 18201–18206. 10.1073/pnas.1407361111 25404306 PMC4280610

[B56] Jensen R. B. Gerdes K. (1995). Programmed cell death in bacteria: proteic plasmid stabilization systems. *Mol. Microbiol.* 17 205–210. 10.1111/j.1365-2958.1995.mmi_17020205.x 7494469

[B57] Jurkin J. Henkel T. Nielsen A. F. Minnich M. Popow J. Kaufmann T. (2014). The mammalian tRNA ligase complex mediates splicing of XBP1 mRNA and controls antibody secretion in plasma cells. *EMBO J.* 33 2922–2936. 10.15252/embj.201490332 25378478 PMC4282640

[B58] Kaufmann G. (2000). Anticodon nucleases. *Trends Biochem. Sci.* 25 70–74. 10.1016/S0968-0004(99)01525-X 10664586

[B59] Khusial P. Plaag R. Zieve G. W. (2005). LSm proteins form heptameric rings that bind to RNA via repeating motifs. *Trends Biochem. Sci.* 30 522–528. 10.1016/j.tibs.2005.07.006 16051491

[B60] Kim N. Huang S. N. Williams J. S. Li Y. C. Clark A. B. Cho J. E. (2011). Mutagenic processing of ribonucleotides in DNA by yeast topoisomerase I. *Science* 332 1561–1564. 10.1126/science.1205016 21700875 PMC3380281

[B61] Kirino Y. Mourelatos Z. (2007). Mouse Piwi-interacting RNAs are 2′-O-methylated at their 3′ termini. *Nat. Struct. Mol. Biol.* 14 347–348. 10.1038/nsmb1218 17384647

[B62] Klassen R. Paluszynski J. P. Wemhoff S. Pfeiffer A. Fricke J. Meinhardt F. (2008). The primary target of the killer toxin from *Pichia acaciae* is tRNA(Gln). *Mol. Microbiol.* 69 681–697. 10.1111/j.1365-2958.2008.06319.x 18532979

[B63] Klassen R. Teichert S. Meinhardt F. (2004). Novel yeast killer toxins provoke S-phase arrest and DNA damage checkpoint activation. *Mol. Microbiol.* 53 263–273. 10.1111/j.1365-2958.2004.04119.x 15225320

[B64] Konarska M. Filipowicz W. Gross H. J. (1982). RNA ligation via 2′-phosphomonoester, 3′5′-phosphodiester linkage: requirement of 2′,3′-cyclic phosphate termini and involvement of a 5′-hydroxyl polynucleotide kinase. *Proc. Natl. Acad. Sci. U.S.A.* 79 1474–1478. 10.1073/pnas.79.5.1474 6280184 PMC345996

[B65] Kurachi K. Davie E. W. Strydom D. J. Riordan J. F. Vallee B. L. (1985). Sequence of the cDNA and gene for angiogenin, a human angiogenesis factor. *Biochemistry* 24 5494–5499. 10.1021/bi00341a032 2866795

[B66] Laneve P. Gioia U. Ragno R. Altieri F. Di Franco C. Santini T. (2008). The tumor marker human placental protein 11 is an endoribonuclease. *J. Biol. Chem.* 283 34712–34719. 10.1074/jbc.M805759200 18936097 PMC3259861

[B67] Leonidas D. D. Shapiro R. Allen S. C. Subbarao G. V. Veluraja K. Acharya K. R. (1999). Refined crystal structures of native human angiogenin and two active site variants: implications for the unique functional properties of an enzyme involved in neovascularisation during tumour growth. *J. Mol. Biol.* 285 1209–1233. 10.1006/jmbi.1998.2378 9918722

[B68] Leonidas D. D. Shapiro R. Subbarao G. V. Russo A. Acharya K. R. (2002). Crystallographic studies on the role of the C-terminal segment of human angiogenin in defining enzymatic potency. *Biochemistry* 41 2552–2562. 10.1021/bi015768q 11851402

[B69] Li S. Lunse C. E. Harris K. A. Breaker R. R. (2015). Biochemical analysis of hatchet self-cleaving ribozymes. *RNA* 21 1845–1851. 10.1261/rna.052522.115 26385510 PMC4604424

[B70] Licht K. Medenbach J. Luhrmann R. Kambach C. Bindereif A. (2008). 3′-cyclic phosphorylation of U6 snRNA leads to recruitment of recycling factor p110 through LSm proteins. *RNA* 14 1532–1538. 10.1261/rna.1129608 18567812 PMC2491463

[B71] Lilley D. M. (2011). Mechanisms of RNA catalysis. *Philos. Trans. R. Soc. Lond. B Biol. Sci.* 366 2910–2917. 10.1098/rstb.2011.0132 21930582 PMC3158914

[B72] Lu J. Esberg A. Huang B. Bystrom A. S. (2008). *Kluyveromyces lactis* gamma-toxin, a ribonuclease that recognizes the anticodon stem loop of tRNA. *Nucleic Acids Res.* 36 1072–1080. 10.1093/nar/gkm1121 18096622 PMC2275089

[B73] Lu J. Huang B. Esberg A. Johansson M. J. Bystrom A. S. (2005). The *Kluyveromyces lactis* gamma-toxin targets tRNA anticodons. *RNA* 11 1648–1654. 10.1261/rna.2172105 16244131 PMC1370851

[B74] Lu Y. Liang F. X. Wang X. (2014). A synthetic biology approach identifies the mammalian UPR RNA ligase RtcB. *Mol. Cell* 55 758–770. 10.1016/j.molcel.2014.06.032 25087875 PMC4156904

[B75] Lund E. Dahlberg J. E. (1992). Cyclic 2′,3′-phosphates and nontemplated nucleotides at the 3′ end of spliceosomal U6 small nuclear RNA’s. *Science* 255 327–330. 10.1126/science.1549778 1549778

[B76] Lyons S. M. Achorn C. Kedersha N. L. Anderson P. J. Ivanov P. (2016). YB-1 regulates tiRNA-induced Stress Granule formation but not translational repression. *Nucleic Acids Res.* 44 6949–6960. 10.1093/nar/gkw418 27174937 PMC5001593

[B77] Maraia R. J. Bayfield M. A. (2006). The La protein-RNA complex surfaces. *Mol. Cell* 21 149–152. 10.1016/j.molcel.2006.01.004 16427005

[B78] Maraia R. J. Intine R. V. (2002). La protein and its associated small nuclear and nucleolar precursor RNAs. *Gene Expr.* 10 41–57. 11868987 PMC5977531

[B79] Mazumder R. Iyer L. M. Vasudevan S. Aravind L. (2002). Detection of novel members, structure-function analysis and evolutionary classification of the 2H phosphoesterase superfamily. *Nucleic Acids Res.* 30 5229–5243. 10.1093/nar/gkf645 12466548 PMC137960

[B80] McCracken D. A. Martin V. J. Stark M. J. Bolen P. L. (1994). The linear-plasmid-encoded toxin produced by the yeast *Pichia acaciae*: characterization and comparison with the toxin of *Kluyveromyces lactis*. *Microbiology* 140(Pt 2), 425–431. 10.1099/13500872-140-2-425 8180706

[B81] Mets T. Lippus M. Schryer D. Liiv A. Kasari V. Paier A. (2017). Toxins MazF and MqsR cleave *Escherichia coli* rRNA precursors at multiple sites. *RNA Biol.* 14 124–135. 10.1080/15476286.2016.1259784 27858580 PMC5270532

[B82] Moll I. Engelberg-Kulka H. (2012). Selective translation during stress in *Escherichia coli*. *Trends Biochem. Sci.* 37 493–498. 10.1016/j.tibs.2012.07.007 22939840 PMC4894542

[B83] Morad I. Chapman-Shimshoni D. Amitsur M. Kaufmann G. (1993). Functional expression and properties of the tRNA(Lys)-specific core anticodon nuclease encoded by *Escherichia coli* prrC. *J. Biol. Chem.* 268 26842–26849. 8262917

[B84] Mroczek S. Dziembowski A. (2013). U6 RNA biogenesis and disease association. *Wiley Interdiscip. Rev. RNA* 4 581–592. 10.1002/wrna.1181 23776162

[B85] Mroczek S. Krwawicz J. Kutner J. Lazniewski M. Kucinski I. Ginalski K. (2012). C16orf57, a gene mutated in poikiloderma with neutropenia, encodes a putative phosphodiesterase responsible for the U6 snRNA 3′ end modification. *Genes Dev.* 26 1911–1925. 10.1101/gad.193169.112 22899009 PMC3435495

[B86] Myllykoski M. Raasakka A. Lehtimaki M. Han H. Kursula I. Kursula P. (2013). Crystallographic analysis of the reaction cycle of 2′,3′-cyclic nucleotide 3′-phosphodiesterase, a unique member of the 2H phosphoesterase family. *J. Mol. Biol.* 425 4307–4322. 10.1016/j.jmb.2013.06.012 23831225 PMC7094350

[B87] Namavar Y. Barth P. G. Kasher P. R. van Ruissen F. Brockmann K. Bernert G. (2011). Clinical, neuroradiological and genetic findings in pontocerebellar hypoplasia. *Brain* 134(Pt 1), 143–156. 10.1093/brain/awq287 20952379 PMC9136852

[B88] Nasr F. Filipowicz W. (2000). Characterization of the *Saccharomyces cerevisiae* cyclic nucleotide phosphodiesterase involved in the metabolism of ADP-ribose 1”,2”-cyclic phosphate. *Nucleic Acids Res.* 28 1676–1683. 10.1093/nar/28.8.1676 10734185 PMC102830

[B89] Nichols N. M. Yue D. (2008). Ribonucleases. *Curr. Protoc. Mol. Biol.* 84 3.13.1–3.13.8. 10.1002/0471142727.mb0313s84 18972385

[B90] Nikolaev Y. Deillon C. Hoffmann S. R. Bigler L. Friess S. Zenobi R. (2010). The leucine zipper domains of the transcription factors GCN4 and c-Jun have ribonuclease activity. *PLoS One* 5:e10765. 10.1371/journal.pone.0010765 20505831 PMC2874015

[B91] Ogawa T. Inoue S. Yajima S. Hidaka M. Masaki H. (2006). Sequence-specific recognition of colicin E5, a tRNA-targeting ribonuclease. *Nucleic Acids Res.* 34 6065–6073. 10.1093/nar/gkl629 16963495 PMC1635277

[B92] Ogawa T. Tomita K. Ueda T. Watanabe K. Uozumi T. Masaki H. (1999). A cytotoxic ribonuclease targeting specific transfer RNA anticodons. *Science* 283 2097–2100. 10.1126/science.283.5410.2097 10092236

[B93] Ohara T. Sakaguchi Y. Suzuki T. Ueda H. Miyauchi K. (2007). The 3′ termini of mouse Piwi-interacting RNAs are 2′-O-methylated. *Nat. Struct. Mol. Biol.* 14 349–350. 10.1038/nsmb1220 17384646

[B94] Paushkin S. V. Patel M. Furia B. S. Peltz S. W. Trotta C. R. (2004). Identification of a human endonuclease complex reveals a link between tRNA splicing and pre-mRNA 3′ end formation. *Cell* 117 311–321. 10.1016/S0092-8674(04)00342-3 15109492

[B95] Peebles C. L. Gegenheimer P. Abelson J. (1983). Precise excision of intervening sequences from precursor tRNAs by a membrane-associated yeast endonuclease. *Cell* 32 525–536. 10.1016/0092-8674(83)90472-5 6186398

[B96] Phizicky E. M. Hopper A. K. (2010). tRNA biology charges to the front. *Genes Dev.* 24 1832–1860. 10.1101/gad.1956510 20810645 PMC2932967

[B97] Pillon M. C. Sobhany M. Borgnia M. J. Williams J. G. Stanley R. E. (2017). Grc3 programs the essential endoribonuclease Las1 for specific RNA cleavage. *Proc. Natl. Acad. Sci. U.S.A.* 114 E5530–E5538. 10.1073/pnas.1703133114 28652339 PMC5514736

[B98] Popow J. Englert M. Weitzer S. Schleiffer A. Mierzwa B. Mechtler K. (2011). HSPC117 is the essential subunit of a human tRNA splicing ligase complex. *Science* 331 760–764. 10.1126/science.1197847 21311021

[B99] Popow J. Schleiffer A. Martinez J. (2012). Diversity and roles of (t)RNA ligases. *Cell. Mol. Life Sci.* 69 2657–2670. 10.1007/s00018-012-0944-2 22426497 PMC3400036

[B100] Puerta-Fernandez E. Romero-Lopez C. Barroso-delJesus A. Berzal-Herranz A. (2003). Ribozymes: recent advances in the development of RNA tools. *FEMS Microbiol. Rev.* 27 75–97. 10.1016/S0168-6445(03)00020-2 12697343

[B101] Roberts G. C. Dennis E. A. Meadows D. H. Cohen J. S. Jardetzky O. (1969). The mechanism of action of ribonuclease. *Proc. Natl. Acad. Sci. U.S.A.* 62 1151–1158. 10.1073/pnas.62.4.1151 5256413 PMC223627

[B102] Roth A. Weinberg Z. Chen A. G. Kim P. B. Ames T. D. Breaker R. R. (2014). A widespread self-cleaving ribozyme class is revealed by bioinformatics. *Nat. Chem. Biol.* 10 56–60. 10.1038/nchembio.1386 24240507 PMC3867598

[B103] Russo N. Shapiro R. Acharya K. R. Riordan J. F. Vallee B. L. (1994). Role of glutamine-117 in the ribonucleolytic activity of human angiogenin. *Proc. Natl. Acad. Sci. U.S.A.* 91 2920–2924. 10.1073/pnas.91.8.2920 8159680 PMC43486

[B104] Salehi-Ashtiani K. Luptak A. Litovchick A. Szostak J. W. (2006). A genomewide search for ribozymes reveals an HDV-like sequence in the human CPEB3 gene. *Science* 313 1788–1792. 10.1126/science.1129308 16990549

[B105] Saville B. J. Collins R. A. (1990). A site-specific self-cleavage reaction performed by a novel RNA in *Neurospora* mitochondria. *Cell* 61 685–696. 10.1016/0092-8674(90)90480-3 2160856

[B106] Saxena S. K. Rybak S. M. Davey R. T. Jr. Youle R. J. Ackerman E. J. (1992). Angiogenin is a cytotoxic, tRNA-specific ribonuclease in the RNase A superfamily. *J. Biol. Chem.* 267 21982–21986. 1400510

[B107] Schifano J. M. Cruz J. W. Vvedenskaya I. O. Edifor R. Ouyang M. Husson R. N. (2016). tRNA is a new target for cleavage by a MazF toxin. *Nucleic Acids Res.* 44 1256–1270. 10.1093/nar/gkv1370 26740583 PMC4756823

[B108] Schifano J. M. Edifor R. Sharp J. D. Ouyang M. Konkimalla A. Husson R. N. (2013). Mycobacterial toxin MazF-mt6 inhibits translation through cleavage of 23S rRNA at the ribosomal A site. *Proc. Natl. Acad. Sci. U.S.A.* 110 8501–8506. 10.1073/pnas.1222031110 23650345 PMC3666664

[B109] Schifano J. M. Vvedenskaya I. O. Knoblauch J. G. Ouyang M. Nickels B. E. Woychik N. A. (2014). An RNA-seq method for defining endoribonuclease cleavage specificity identifies dual rRNA substrates for toxin MazF-mt3. *Nat. Commun.* 5:3538. 10.1038/ncomms4538 24709835 PMC4090939

[B110] Schillewaert S. Wacheul L. Lhomme F. Lafontaine D. L. (2012). The evolutionarily conserved protein Las1 is required for pre-rRNA processing at both ends of ITS2. *Mol. Cell. Biol.* 32 430–444. 10.1128/MCB.06019-11 22083961 PMC3255765

[B111] Schutz K. Hesselberth J. R. Fields S. (2010). Capture and sequence analysis of RNAs with terminal 2′,3′-cyclic phosphates. *RNA* 16 621–631. 10.1261/rna.1934910 20075163 PMC2822926

[B112] Scott W. G. Klug A. (1996). Ribozymes: structure and mechanism in RNA catalysis. *Trends Biochem. Sci.* 21 220–224. 10.1016/S0968-0004(96)80019-3 8744356

[B113] Sekiguchi J. Shuman S. (1997). Site-specific ribonuclease activity of eukaryotic DNA topoisomerase I. *Mol. Cell* 1 89–97. 10.1016/S1097-2765(00)80010-6 9659906

[B114] Serganov A. Patel D. J. (2007). Ribozymes, riboswitches and beyond: regulation of gene expression without proteins. *Nat. Rev. Genet.* 8 776–790. 10.1038/nrg2172 17846637 PMC4689321

[B115] Shapiro R. Riordan J. F. Vallee B. L. (1986). Characteristic ribonucleolytic activity of human angiogenin. *Biochemistry* 25 3527–3532. 10.1021/bi00360a008 2424496

[B116] Shchepachev V. Wischnewski H. Missiaglia E. Soneson C. Azzalin C. M. (2012). Mpn1, mutated in poikiloderma with neutropenia protein 1, is a conserved 3′-to-5′ RNA exonuclease processing U6 small nuclear RNA. *Cell Rep.* 2 855–865. 10.1016/j.celrep.2012.08.031 23022480

[B117] Sheng J. Xu Z. (2016). Three decades of research on angiogenin: a review and perspective. *Acta Biochim. Biophys. Sin.* 48 399–410. 10.1093/abbs/gmv131 26705141 PMC4888354

[B118] Shigematsu M. Honda S. Kirino Y. (2018). Dumbbell-PCR for discriminative quantification of a small RNA variant. *Methods Mol. Biol.* 1680 65–73. 10.1007/978-1-4939-7339-2_4 29030841

[B119] Shigematsu M. Kirino Y. (2015). tRNA-derived short non-coding RNA as interacting partners of argonaute proteins. *Gene Regul. Syst. Biol.* 9 27–33. 10.4137/GRSB.S29411 26401098 PMC4567038

[B120] Shih I. H. Been M. D. (2002). Catalytic strategies of the hepatitis delta virus ribozymes. *Annu. Rev. Biochem.* 71 887–917. 10.1146/annurev.biochem.71.110601.135349 12045114

[B121] Shinya S. Kadokura H. Imagawa Y. Inoue M. Yanagitani K. Kohno K. (2011). Reconstitution and characterization of the unconventional splicing of XBP1u mRNA in vitro. *Nucleic Acids Res.* 39 5245–5254. 10.1093/nar/gkr132 21398633 PMC3130286

[B122] Shuman S. (1998). Polynucleotide ligase activity of eukaryotic topoisomerase I. *Mol. Cell* 1 741–748. 10.1016/S1097-2765(00)80073-8 9660957

[B123] Sidrauski C. Walter P. (1997). The transmembrane kinase Ire1p is a site-specific endonuclease that initiates mRNA splicing in the unfolded protein response. *Cell* 90 1031–1039. 10.1016/S0092-8674(00)80369-4 9323131

[B124] Sporn M. B. Lazarus H. M. Smith J. M. Henderson W. R. (1969). Studies on nuclear exoribonucleases. 3. Isolation and properties of the enzyme from normal and malignant tissues of the mouse. *Biochemistry* 8 1698–1706. 10.1021/bi00832a053 5805304

[B125] Stark M. J. Boyd A. (1986). The killer toxin of *Kluyveromyces lactis*: characterization of the toxin subunits and identification of the genes which encode them. *EMBO J.* 5 1995–2002. 10.1002/j.1460-2075.1986.tb04455.x 3758030 PMC1167069

[B126] Strydom D. J. Fett J. W. Lobb R. R. Alderman E. M. Bethune J. L. Riordan J. F. (1985). Amino acid sequence of human tumor derived angiogenin. *Biochemistry* 24 5486–5494. 10.1021/bi00341a031 2866794

[B127] Tanaka N. Chakravarty A. K. Maughan B. Shuman S. (2011a). Novel mechanism of RNA repair by RtcB via sequential 2′,3′-cyclic phosphodiesterase and 3′-Phosphate/5′-hydroxyl ligation reactions. *J. Biol. Chem.* 286 43134–43143. 10.1074/jbc.M111.302133 22045815 PMC3234866

[B128] Tanaka N. Meineke B. Shuman S. (2011b). RtcB, a novel RNA ligase, can catalyze tRNA splicing and HAC1 mRNA splicing in vivo. *J. Biol. Chem.* 286 30253–30257. 10.1074/jbc.C111.274597 21757685 PMC3162383

[B129] Tanaka N. Shuman S. (2011). RtcB is the RNA ligase component of an *Escherichia coli* RNA repair operon. *J. Biol. Chem.* 286 7727–7731. 10.1074/jbc.C111.219022 21224389 PMC3048659

[B130] Tello-Montoliu A. Patel J. V. Lip G. Y. (2006). Angiogenin: a review of the pathophysiology and potential clinical applications. *J. Thromb. Haemost.* 4 1864–1874. 10.1111/j.1538-7836.2006.01995.x 16961595

[B131] Telonis A. G. Loher P. Honda S. Jing Y. Palazzo J. Kirino Y. (2015). Dissecting tRNA-derived fragment complexities using personalized transcriptomes reveals novel fragment classes and unexpected dependencies. *Oncotarget* 6 24797–24822. 10.18632/oncotarget.4695 26325506 PMC4694795

[B132] Temmel H. Muller C. Sauert M. Vesper O. Reiss A. Popow J. (2017). The RNA ligase RtcB reverses MazF-induced ribosome heterogeneity in *Escherichia coli*. *Nucleic Acids Res.* 45 4708–4721. 27789694 10.1093/nar/gkw1018 PMC5416887

[B133] Thiyagarajan N. Ferguson R. Subramanian V. Acharya K. R. (2012). Structural and molecular insights into the mechanism of action of human angiogenin-ALS variants in neurons. *Nat. Commun.* 3:1121. 10.1038/ncomms2126 23047679 PMC3493651

[B134] Thompson J. E. Raines R. T. (1994). Value of general Acid-base catalysis to ribonuclease a. *J. Am. Chem. Soc.* 116 5467–5468. 10.1021/ja00091a060 21391696 PMC3056461

[B135] Tomita K. Ogawa T. Uozumi T. Watanabe K. Masaki H. (2000). A cytotoxic ribonuclease which specifically cleaves four isoaccepting arginine tRNAs at their anticodon loops. *Proc. Natl. Acad. Sci. U.S.A.* 97 8278–8283. 10.1073/pnas.140213797 10880568 PMC26938

[B136] Torchia C. Takagi Y. Ho C. K. (2008). Archaeal RNA ligase is a homodimeric protein that catalyzes intramolecular ligation of single-stranded RNA and DNA. *Nucleic Acids Res.* 36 6218–6227. 10.1093/nar/gkn602 18829718 PMC2577357

[B137] Trotta C. R. Miao F. Arn E. A. Stevens S. W. Ho C. K. Rauhut R. (1997). The yeast tRNA splicing endonuclease: a tetrameric enzyme with two active site subunits homologous to the archaeal tRNA endonucleases. *Cell* 89 849–858. 10.1016/S0092-8674(00)80270-6 9200603

[B138] Unterholzner S. J. Poppenberger B. Rozhon W. (2013). Toxin-antitoxin systems: biology, identification, and application. *Mob. Genet. Elements* 3:e26219. 10.4161/mge.26219 24251069 PMC3827094

[B139] Urano F. Bertolotti A. Ron D. (2000). IRE1 and efferent signaling from the endoplasmic reticulum. *J. Cell Sci.* 113(Pt 21), 3697–3702. 11034898 10.1242/jcs.113.21.3697

[B140] Vesper O. Amitai S. Belitsky M. Byrgazov K. Kaberdina A. C. Engelberg-Kulka H. (2011). Selective translation of leaderless mRNAs by specialized ribosomes generated by MazF in *Escherichia coli*. *Cell* 147 147–157. 10.1016/j.cell.2011.07.047 21944167 PMC4894548

[B141] Ward W. L. Plakos K. DeRose V. J. (2014). Nucleic acid catalysis: metals, nucleobases, and other cofactors. *Chem. Rev.* 114 4318–4342. 10.1021/cr400476k 24730975 PMC4002065

[B142] Weinberg Z. Kim P. B. Chen T. H. Li S. Harris K. A. Lunse C. E. (2015). New classes of self-cleaving ribozymes revealed by comparative genomics analysis. *Nat. Chem. Biol.* 11 606–610. 10.1038/nchembio.1846 26167874 PMC4509812

[B143] Winkler W. C. Nahvi A. Roth A. Collins J. A. Breaker R. R. (2004). Control of gene expression by a natural metabolite-responsive ribozyme. *Nature* 428 281–286. 10.1038/nature02362 15029187

[B144] Yajima S. Inoue S. Ogawa T. Nonaka T. Ohsawa K. Masaki H. (2006). Structural basis for sequence-dependent recognition of colicin E5 tRNase by mimicking the mRNA-tRNA interaction. *Nucleic Acids Res.* 34 6074–6082. 10.1093/nar/gkl729 17099236 PMC1669751

[B145] Yamasaki S. Ivanov P. Hu G. F. Anderson P. (2009). Angiogenin cleaves tRNA and promotes stress-induced translational repression. *J. Cell Biol.* 185 35–42. 10.1083/jcb.200811106 19332886 PMC2700517

[B146] Yang Z. Ebright Y. W. Yu B. Chen X. (2006). HEN1 recognizes 21-24 nt small RNA duplexes and deposits a methyl group onto the 2′ OH of the 3′ terminal nucleotide. *Nucleic Acids Res.* 34 667–675. 10.1093/nar/gkj474 16449203 PMC1356533

[B147] Yarmolinsky M. B. (1995). Programmed cell death in bacterial populations. *Science* 267 836–837. 10.1126/science.7846528 7846528

[B148] Yoshihisa T. (2014). Handling tRNA introns, archaeal way and eukaryotic way. *Front. Genet.* 5:213. 10.3389/fgene.2014.00213 25071838 PMC4090602

[B149] Yoshinari S. Liu Y. Gollnick P. Ho C. K. (2017). Cleavage of 3′-terminal adenosine by archaeal ATP-dependent RNA ligase. *Sci. Rep.* 7:11662. 10.1038/s41598-017-11693-0 28912583 PMC5599603

[B150] Zaug A. J. Linger J. Cech T. R. (1996). Method for determining RNA 3′ ends and application to human telomerase RNA. *Nucleic Acids Res.* 24 532–533. 10.1093/nar/24.3.532 8602368 PMC145649

[B151] Zhang K. Kaufman R. J. (2004). Signaling the unfolded protein response from the endoplasmic reticulum. *J. Biol. Chem.* 279 25935–25938. 10.1074/jbc.R400008200 15070890

[B152] Zhang Y. Zhang J. Hara H. Kato I. Inouye M. (2005a). Insights into the mRNA cleavage mechanism by MazF, an mRNA interferase. *J. Biol. Chem.* 280 3143–3150. 10.1074/jbc.M411811200 15537630

[B153] Zhang Y. Zhu L. Zhang J. Inouye M. (2005b). Characterization of ChpBK, an mRNA interferase from *Escherichia coli*. *J. Biol. Chem.* 280 26080–26088. 10.1074/jbc.M502050200 15901733

[B154] Zhang Y. Zhang J. Hoeflich K. P. Ikura M. Qing G. Inouye M. (2003). MazF cleaves cellular mRNAs specifically at ACA to block protein synthesis in *Escherichia coli*. *Mol. Cell* 12 913–923. 10.1016/S1097-2765(03)00402-7 14580342

[B155] Zhao C. Tolkach Y. Schmidt D. Muders M. Kristiansen G. Muller S. C. (2018). tRNA-halves are prognostic biomarkers for patients with prostate cancer. *Urol. Oncol.* 36 503.e1–503.e7. 10.1016/j.urolonc.2018.08.003 30209018

[B156] Zhelkovsky A. M. McReynolds L. A. (2014). Polynucleotide 3′-terminal phosphate modifications by RNA and DNA ligases. *J. Biol. Chem.* 289 33608–33616. 10.1074/jbc.M114.612929 25324547 PMC4246112

[B157] Zinder J. C. Wasmuth E. V. Lima C. D. (2016). Nuclear RNA exosome at 3.1 A reveals substrate specificities, RNA paths, and allosteric inhibition of Rrp44/Dis3. *Mol. Cell* 64 734–745. 10.1016/j.molcel.2016.09.038 27818140 PMC5115963

